# Economic Burden of Recurrence Among Resected Medicare Patients With Early Stage NSCLC

**DOI:** 10.1016/j.jtocrr.2023.100487

**Published:** 2023-02-25

**Authors:** Jay M. Lee, Rongrong Wang, Ann Johnson, Sarika Ogale, Matthew Kent, Janet S. Lee

**Affiliations:** a Division of Thoracic Surgery, David Geffen School of Medicine at University of California, Los Angeles (UCLA), Los Angeles, California; b US Medical Affairs, Genentech Inc., South San Francisco, California; c RWE-Analytics, Genesis Research, Hoboken, New Jersey

**Keywords:** Early non–small cell lung cancer, Health economics, Healthcare resource utilization, Costs, Immunotherapy

## Abstract

**Introduction:**

Patients with early NSCLC (eNSCLC) who experience recurrence are associated with worse survival outcomes, but the economic burden of recurrence is not well characterized. This study evaluated the incremental health care resource utilization and costs of recurrence in Medicare patients with resected eNSCLC.

**Methods:**

This retrospective observational study used Surveillance, Epidemiology, and End Results cancer registry data linked with Medicare claims. Eligible patients were 65 years and older with newly diagnosed NSCLC stages IB to IIIA (American Joint Committee on Cancer Staging Manual, seventh edition) and surgery between January 2010 and December 2017. Continuous enrollment criteria were applied to ensure appropriate data capture. Per patient per month (PPPM) health care resource utilization and all-cause direct costs were compared for patients with versus without recurrence, which was identified from claims data using diagnosis, procedure, or drug codes. Patients were matched (1:1) using exact matching on cancer stage and treatment, and propensity score matching on other characteristics.

**Results:**

In total, 2035 (44%) out of 4595 patients had evidence of recurrence. After matching, 1494 patients were included in each cohort. Patients with recurrence had a significantly higher number of inpatient visits (+0.25 PPPM), outpatient visits (+1.10 PPPM), physician services (+3.70 PPPM), and emergency department (ED) visits (+0.25 PPPM; all *p* < 0.001). The average follow-up PPPM cost in the recurrence cohort was U.S. dollars $7437 and $1118 in the no-recurrence cohort, resulting in a difference of $6319 PPPM (*p* < 0.001) with inpatient costs as the largest contributor.

**Conclusions:**

On the basis of a real-world population, the recurrence among patients with resected eNSCLC is associated with increased health care resource utilization and costs.

## Introduction

NSCLC accounts for more than 75% of all lung cancer cases in the United States,^1^ and more than half of the patients with NSCLC are diagnosed with stage I, stage II, or resectable stage III disease, known as early NSCLC (eNSCLC).^2^ The expected 5-year survival of patients with eNSCLC deteriorates rapidly with advanced disease, from 92% for patients with resected stage IA to 36% for those with stage IIIA disease.^3^^,^^4^

Surgical resection is the preferred approach for operable patients with resectable eNSCLC, and adjuvant chemotherapy was historically the standard of care for metastatic lymph node disease.^5^ However, the overall survival benefit observed in clinical trials of adjuvant chemotherapy in this setting was modest,^6^ and recurrence rates were high for these patients.^6^^,^^7^ Survival outcomes are known to worsen with recurrence,^3^ but the corresponding economic burden in terms of real-world health care resource utilization (HCRU) and costs among patients with resected eNSCLC with recurrence has not been well characterized.

Estimating the economic burden of recurrence has several important implications for health economic evaluations and policy decisions, and is essential for understanding the value of new treatment options for patients with resected eNSCLC. Newer treatment options such as adjuvant or neoadjuvant use of immune checkpoint inhibitors (ICIs) or targeted therapies have been found to reduce the risk of recurrence, which may decrease the economic burden of eNSCLC.^8^^,^^9^ Significant disease-free survival was shown for stage II to IIIA patients with adjuvant atezolizumab after a median follow-up of 32.2 months in the IMpower010 trial (*p* = 0.0039) (NCT02486718)^9^ and with adjuvant osimertinib after a median follow-up of 24 months in the ADAURA trial (*p* < 0.001) (NCT02511106).^8^ Adjuvant pembrolizumab has also improved disease-free survival for patients with stage I to IIIA NSCLC after resection regardless of programmed death-ligand 1 expression in the KEYNOTE-091 trial (NCT02504372).^10^ Nivolumab is approved with platinum-doublet chemotherapy as neoadjuvant treatment for patients with resectable NSCLC on the basis of findings from the phase 3 CheckMate 816 trial (NCT02998529).^11^^,^^12^ Other phase 3 trials of neoadjuvant chemotherapy plus ICIs and adjuvant ICI are ongoing for use in patients with eNSCLC.^13^

Because the median age at lung cancer diagnosis is 70 years in the United States,^14^ the health economics of eNSCLC treatment is particularly relevant to the Medicare population. Furthermore, previous research has revealed that a substantial proportion of Medicare patients with eNSCLC do not receive adjuvant treatment in the real-world setting, suggesting an unmet therapeutic need in the adjuvant setting for this patient population.^15^ Whereas patients with recurrence would be expected to incur higher costs than those without recurrence, the magnitude of the cost difference is not reported in the literature but is essential for accurate health economic evaluations that inform health policy and treatment access decisions. Thus, this study evaluated the incremental HCRU and costs associated with recurrence identified largely on the basis of treatment proxies among Medicare patients with resected eNSCLC.

## Materials and Methods

### Study Design

**Figure 1:**
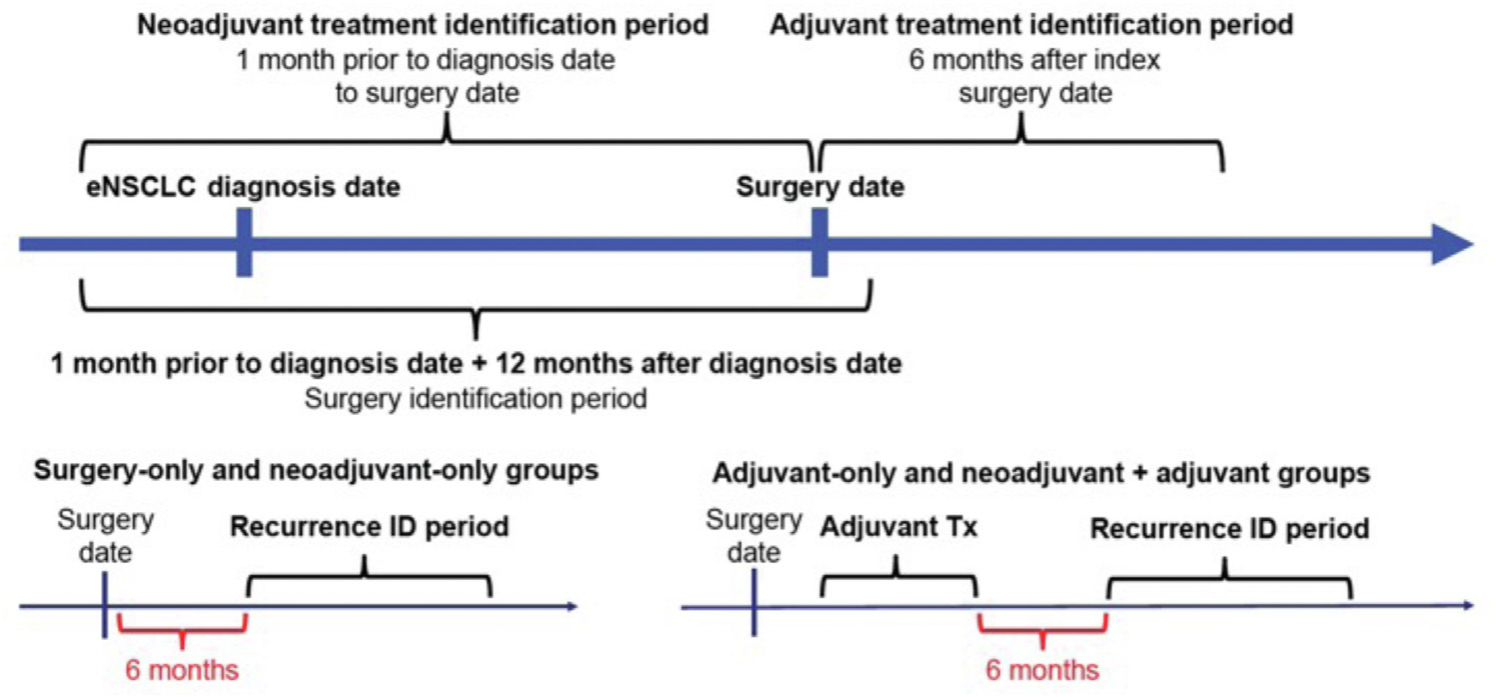
Study design. eNSCLC, early NSCLC; ID, identification; Tx, treatment.

This retrospective observational cohort study used Surveillance, Epidemiology, and End Results (SEER) cancer registry data linked with Medicare beneficiary claims data (National Cancer Institute; healthcaredelivery.cancer.gov/seermedicare/). Eligible patients had to be aged 65 years or older with newly diagnosed NSCLC (International Classification of Diseases for Oncology [ICD-O] codes 8000-8040, 8046-9989) stages IB to IIIA (American Joint Committee on Cancer [AJCC] Staging Manual, seventh edition)^16^ who underwent surgery between January 2010 and December 2017. Patients were required to have continuous enrollment in Medicare parts A, B, and D for at least 7 months before and at least 12 months after diagnosis to ensure adequate capture of baseline characteristics and surgery, and during the 6 months after the surgery date to adequately capture adjuvant treatment (Fig. 1). Once patients were identified with adjuvant or neoadjuvant treatment, additional continuous enrollment criteria were applied to ensure adequate capture of recurrence. Specifically, they were required to have at least 6 months of continuous enrollment after the surgery date for patients who only had surgery or only neoadjuvant treatment. For patients who had adjuvant treatment only or received neoadjuvant plus adjuvant treatment, at least 6 months of continuous enrollment were required after the end of the first-line adjuvant therapy. Continuous enrollment in Medicare parts A, B, and D for at least 1 month after the (pseudo) recurrence date (defined below) was also required. Finally, patients were excluded if they were identified with SCLC (ICD-O codes 8041-8045), neuroendocrine or carcinoid tumors (ICD-O codes 8240-8246, 8249), large cell carcinoma (ICD-O codes 8012-8014), or missing diagnosis or staging information. Large cell carcinomas are a type of neuroendocrine carcinoma, which were excluded from this study because of their inherent differences in clinical behavior and management compared with other non-SCLC. Patients enrolled in a health maintenance organization, Veterans Affairs, or military hospital during any of the previously described study periods were excluded because of missing claims data.

### Study Cohorts

Two study cohorts were identified, comprising patients with and without recurrence. Given that recurrence is not directly captured in the SEER database, an algorithm was applied to indirectly identify recurrence from claims data, largely on the basis of the receipt of treatment. The definition of recurrence was adapted from that of Hassett et al.^17^ with a clinical expert consultation. Recurrence, which includes both locoregional and metastatic, was defined using diagnosis and treatment codes as evidence of secondary malignant neoplasm (excluding lung), surgery, radiation, chemotherapy, immunotherapy, or targeted therapy from 6 months after surgery or end of adjuvant treatment until the end of follow-up (end of enrollment, end of study period, or death) (Fig. 1). Consistent with Hassett et al.,^17^ the code for secondary malignant neoplasms of the lung was excluded as an indicator of recurrence because it is impossible to determine whether it refers to original cancer, recurrent lung cancer, or new primary lung cancer. Treatment definitions are provided in the Supplementary Materials (Supplementary Table 1). Recurrence rates derived from the incremental adaptation of the published definition were benchmarked against literature estimates to identify the most appropriate and robust definition of recurrence rate (Supplementary Table 2).

### Outcomes

Patient demographic and clinical characteristics included age at diagnosis, sex, race or ethnicity, income category, college education, Charlson Comorbidity Index score,^18^ derived AJCC stage (AJCC Cancer Staging Manual, seventh edition), tumor grade, histology, lymph node positivity, and surgical and perioperative systemic treatment (neoadjuvant, adjuvant, or both). Income and college education were on the basis of the U.S. Census tract aggregate data and not patient-level data.

HCRU and all-cause direct health care costs were evaluated starting from 2 months before the recurrence date until the end of follow-up. The recurrence date was on the basis of the date of the first qualifying event among patients with recurrence. Because the study was designed to measure postrecurrence costs, a “pseudorecurrence” date was established for patients who did not experience recurrence. Among nonrecurrence patients, pseudorecurrence dates were assigned on the basis of the interval between the surgery or adjuvant treatment end date and recurrence date for the matched recurrence patient (see the matching algorithm in the Statistical Analysis section). For instance, if a patient in the surgery-only cohort experienced recurrence 90 days after surgery, the matched nonrecurrence patient was assigned a pseudorecurrence date 90 days after their surgery date.

HCRU included the number of inpatient, outpatient, physician office, and ED visits per patient per month (PPPM). All-cause direct health care costs included Medicare-reimbursed amounts and out-of-pocket payments PPPM for inpatient and outpatient visits, pharmacy costs, and other encounters. Inpatient services included hospital care covered by Medicare part A, outpatient services included diagnostic and treatment services covered by Medicare part B, pharmacy costs included prescription drugs covered by Medicare part D, and other encounters included durable medical equipment, hospice care, home health, and physician services. Costs were adjusted for inflation to 2021 U.S. dollars using the medical care component of the Consumer Price Index.

### Statistical Analysis

Descriptive statistics were used to summarize patient characteristics, HCRU, and costs. Means (SD^19^) were reported for continuous variables, and percentages were reported for binary and categorical variables. Patients with recurrence were matched 1:1 with patients without recurrence using exact matching on cancer stage and treatment (surgery only, adjuvant only, neoadjuvant only, adjuvant + neoadjuvant). Propensity score matching (PSM) using the nearest neighbor approach was performed on other patient characteristics of age, sex, race or ethnicity, Charlson Comorbidity Index score, histology, lymph node positivity, and tumor grade. Welch’s two-sample *t* tests (with 95% confidence intervals [CI]) assessed differences in PPPM HCRU and costs between patients in the matched recurrence and no-recurrence groups. As a sensitivity analysis, the change in HCRU and costs before and after the recurrence event was evaluated among patients with recurrence, in which each patient served as their own control. The time between the surgery date and 2 months before the recurrence date was defined as the prerecurrence period; the postrecurrence period was defined as the time between the end of the prerecurrence period and the end of enrollment, end of the study period, or death, whichever occurred first. All analyses were conducted using RStudio Team 2020 (www.rstudio.com).

## Results

**Figure 2:**
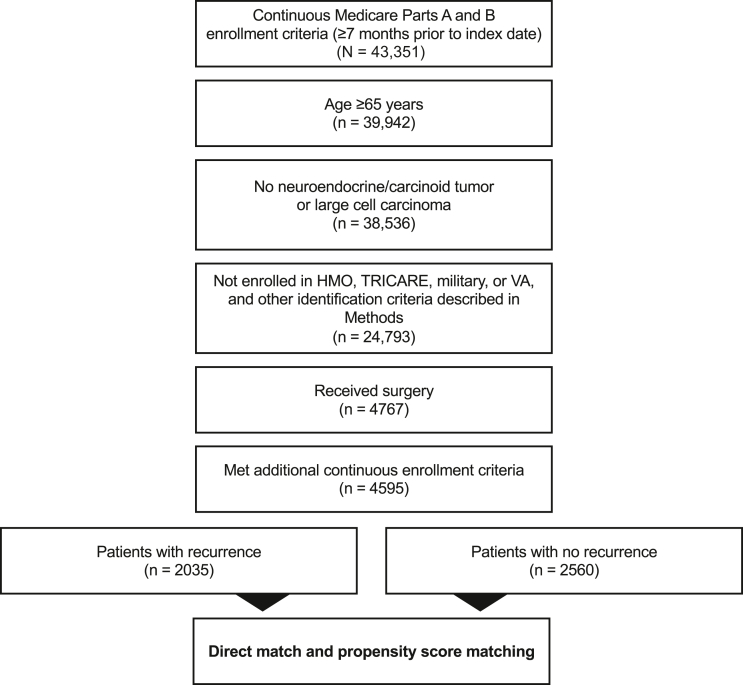
Patient attrition. Recurrence on the basis of definition 2 in Supplementary Table 2. HMO, health maintenance organization; VA, Veterans’ Affairs.

A total of 4595 Medicare patients aged 65 years or older with eNSCLC and surgical resection met the eligibility criteria, of whom 2035 (44%) had evidence of recurrence (Fig. 2). Recurrence rates on the basis of definition 2 (Supplementary Table 2) most closely approximated the published benchmarks,^6^^,^^7^^,^^20^, ^21^, ^22^ provided a robust patient cohort, and were therefore used in this study to identify recurrence. Thus, the term “recurrence” as identified in this study is largely-treatment based recurrence.

Before matching, patient demographics and clinical characteristics were largely similar between study cohorts, but patients with recurrence were more likely to have more advanced tumor stage and grade, histology, positive lymph nodes, and receive adjuvant treatment (Table 1). After PSM, 1494 patients were included in the recurrence and no-recurrence cohorts (Fig. 2). All post-PSM characteristics were similar between cohorts; the mean age was 74 years, 44% to 46% were men, and 87% were White. Of the post-PSM population, 37% had stage IB disease, 38% had stage II disease, and 25% had stage IIIA disease (Table 1).

**Table 1:** Prematching and Postmatching Patient Characteristics

Characteristics	Prematching Groups			Postmatching Groups		
	Recurrence (n = 2035)	No Recurrence (n = 2560)	SMD	Recurrence (n = 1494)	No Recurrence (n = 1494)	SMD
Age at diagnosis, mean (SD), y	74.0 (5.59)	73.9 (5.51)	0.009	73.9 (5.56)	74.0 (5.54)	0.011
Sex, male, n (%)	930 (46)	1136 (44)	0.027	692 (46)	654 (44)	0.051
Race or ethnicity, n (%)			0.041			0.026
White	1764 (87)	2235 (87)		1294 (87)	1301 (87)	
Black	126 (6)	161 (6)		98 (7)	95 (6)	
Asian	136 (7)	149 (6)		95 (6)	89 (6)	
Native American	a	a		a	a	
Missing	a	a		a	a	
Median income, n (%)^b^			0.064			0.053
≤$40,502	433 (21)	557 (22)		303 (20)	332 (22)	
$40,503-$56,178	472 (23)	641 (25)		368 (25)	354 (24)	
$56,179-$79,560	570 (28)	651 (25)		417 (28)	398 (27)	
>$79,560	a	a		a	a	
Missing	a	a		a	a	
College education, n (%)^b^			0.068			0.011
≤14%	418 (21)	546 (21)		310 (21)	311 (21)	
15%-25%	565 (28)	682 (27)		410 (27)	403 (27)	
26%-42%	484 (24)	670 (26)		368 (25)	369 (25)	
>42%	a	a		a	a	
Missing	a	a		a	a	
Charlson Comorbidity Index score at baseline, mean (SD)	2.06 (1.83)	2.10 (1.88)	0.023	2.14 (1.88)	2.03 (1.82)	0.056
Tumor stage (AJCC, seventh edition), n (%)			**0.379**			<0.001
IB	689 (34)	1289 (50)		559 (37)	559 (37)	
II	704 (35)	804 (31)		564 (38)	564 (38)	
IIIA	642 (32)	467 (18)		371 (25)	371 (25)	
Tumor grade, n (%)			**0.171**			0.040
1	173 (9)	340 (13)		135 (9)	147 (10)	
2	887 (44)	1133 (44)		661 (44)	673 (45)	
3	779 (38)	848 (33)		561 (38)	547 (37)	
4	17 (1)	26 (1)		14 (1)	12 (1)	
Missing	179 (9)	213 (8)		123 (8)	115 (8)	
Histology, n (%)			**0.146**			0.040
Adenocarcinoma, NOS	880 (43)	942 (37)		606 (41)	588 (39)	
Squamous cell carcinoma, NOS	570 (28)	864 (34)		456 (31)	484 (32)	
Other	585 (29)	754 (30)		432 (29)	422 (28)	
Positive lymph node resection, n (%)			**0.355**			0.089
No	1088 (54)	1799 (70)		855 (57)	915 (61)	
Yes	778 (38)	602 (24)		511 (34)	475 (32)	
No nodes examined	a	a		a	a	
Unknown	a	a		a	a	
Treatment cohort, n (%)			**0.370**			<0.001
Surgery only	851 (42)	1504 (59)		685 (46)	685 (46)	
Neoadjuvant only	87 (4)	120 (5)		75 (5)	75 (5)	
Adjuvant only	933 (46)	840 (33)		666 (45)	666 (45)	
Neoadjuvant + adjuvant	164 (8)	96 (4)		68 (5)	68 (5)	

*Note:* SMD less than 0.1 was used to determine whether the covariate was considered balanced between groups (boldfaced type indicates SMD >0.1).

Recurrence on the basis of definition 2 in Supplementary Table 2.

AJCC, AJCC Cancer Staging Manual, seventh edition; NOS, not otherwise specified; SMD, standardized mean difference.

a Suppressed cell values (0-10 patients) according to the Centers for Medicare and Medicaid Services Cell Size Suppression Policy (https://resdac.org/articles/cms-cell-size-suppression-policy).

b On the basis of the U.S. Census tract aggregate data, not patient-level data.

### Health Care Resource Utilization

Patients with recurrence had significantly greater HCRU than matched patients without recurrence across all types of healthcare services. On average, the recurrence cohort had more inpatient visits (+0.25 PPPM), outpatient visits (+1.10 PPPM), physician services (+3.70 PPPM), and ED visits (+0.25 PPPM) than the no-recurrence cohort (all *p* < 0.001) (Table 2). Findings were similar in matched cohorts of patients with stage II to IIIA disease (Supplementary Table 3).

**Table 2:** HCRU in Matched Patients With eNSCLC With or Without Recurrence

HCRU, Mean (SD), PPPM	Recurrence (n = 1494)	No recurrence (n = 1494)	Difference (95% CI)	*p* Value
Inpatient visit claims	0.35 (0.51)	0.10 (0.15)	0.25 (0.22–0.28)	<0.001
Outpatient visit claims	1.73 (1.62)	0.58 (0.57)	1.10 (1.10–1.20)	<0.001
Physician services claims	6.00 (4.90)	2.30 (1.90)	3.70 (3.40–3.90)	<0.001
ED visit claims	0.36 (0.47)	0.11 (0.16)	0.25 (0.22–0.27)	<0.001

*Note:* Recurrence on the basis of definition 2 in Supplementary Table 2.

CI, confidence interval; ED, emergency department; eNSCLC, early NSCLC; HCRU, health care resource utilization; PPPM, per patient per month.

### Costs of Recurrence

**Figure 3:**
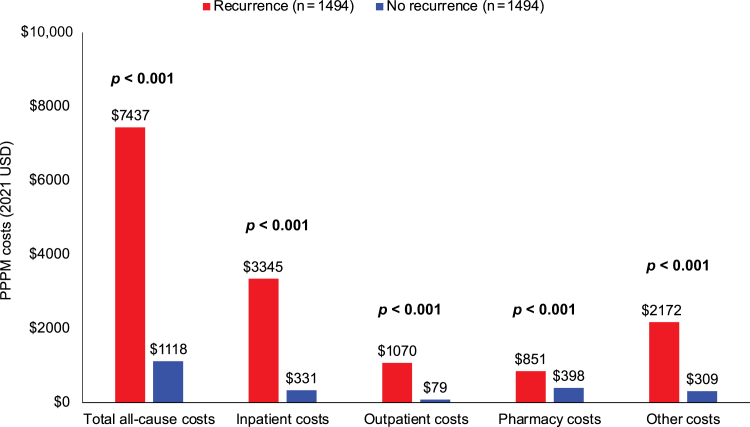
All-cause health care costs in matched patients with eNSCLC with or without recurrence. Inpatient services included hospital care covered by Medicare Part A; outpatient services included diagnostic and treatment services covered by Medicare Part B; pharmacy costs included prescription drugs covered by Medicare Part D; other services included durable medical equipment, hospice care, home health, and physician services. Recurrence on the basis of definition 2 in Supplementary Table 2. eNSCLC, early NSCLC; PPPM, per patient per month; USD, U.S. dollars.

The total mean all-cause health care costs were $6319 PPPM higher (95% CI: $5592–$7047) (*p* < 0.001) for the recurrence cohort than for the no-recurrence cohort over a median follow-up of 14 and 32 months, respectively (Fig. 3). Each component of the total health care service costs was significantly higher for the recurrence cohort, including costs related to inpatient visits (+$3013 PPPM), outpatient visits (+$990 PPPM), pharmacy costs (+$453 PPPM), and other costs (+$1863 PPPM) (all *p* < 0.001) (Fig. 3).

### Sensitivity Analysis: Prerecurrence Versus Postrecurrence HCRU and Costs

A total of 2035 patients with recurrence were included in the analysis of HCRU and costs before and after their recurrence. Total mean all-cause HCRU increased from 5.2 to 9.6 claims PPPM in the pre- versus postrecurrence periods (*p* < 0.001). Postrecurrence HCRU was higher for inpatient visits (+0.26 claims PPPM), outpatient visits (+0.85 claims PPPM), physician services (+3.10 claims PPPM), and ED visits (+0.18 claims PPPM) (Supplementary Table 4).

Similarly, the total mean all-cause health care costs were $4265 PPPM higher in the postrecurrence period (95% CI: $3736–$4794) (*p* < 0.001). All component service costs were significantly higher in the postrecurrence period (all *p* < 0.001) (Supplementary Fig. 5) compared with the prerecurrence period, as observed in the primary analysis comparing costs between patients with versus without recurrence.

## Discussion

This retrospective observational study evaluated the incremental economic burden of recurrence and exhibited substantially greater real-world HCRU and costs associated with recurrence among Medicare patients with resected stage IB to IIIA NSCLC. The total all-cause health care costs were more than six times higher for patients with recurrence than for matched patients without recurrence. The sensitivity analysis was consistent with primary findings in that the incremental HCRU and direct health care cost estimates associated with recurrence were similar in magnitude to those in the primary analysis.

The results from this study are consistent with the published literature. Cai et al.^23^ reported similar HCRU among resected patients with stage II to IIIB NSCLC from the U.S. Oncology Network clinics, in which patients experiencing relapses had considerably more mean ED visits per month (0.10 versus 0.03) and mean hospitalizations per month (0.20 versus 0.05) than those without relapses, respectively, although costs to the payer were not reported. However, costs were not evaluated, and to our knowledge, our study is the first to report real-world costs in addition to HCRU in this patient population.

The results of this study may help health care stakeholders to accurately characterize the value of new treatments in eNSCLC and support evidence-based policy decisions related to the management of these patients. This work is of particular relevance as the use of adjuvant systemic therapies increases after the U.S. approval of the first immunotherapy of adjuvant atezolizumab after platinum-based chemotherapy for resected patients with stage II to IIIA NSCLC whose tumors have programmed death-ligand 1 expression at least 1% of tumor cells.^9^^,^^24^

These findings should be interpreted with consideration of the study's strengths and limitations. The SEER-Medicare linked data set provided robust medical and insurance claims information to inform the payer’s perspective on the incremental economic burden of recurrence in this population. However, these findings may not necessarily be generalizable to a non-Medicare population in the United States or other countries owing to differences in patient characteristics and health system factors that may influence patterns of care. Another limitation of this study is that recurrence was indirectly identified on the basis of insurance claims data and not formally validated such as with medical record abstraction, which may have led to potential misclassification as many elderly patients may not be offered or accept treatment after recurrence because of patient preference or declining health status. As such, the incremental costs of recurrence could potentially be higher in the commercial population; future research should be conducted in other payer populations. The PSM approach was successful in providing a well-matched population of patients with versus without recurrence; however, PSM does not control for differences in unobservable variables or characteristics, which could potentially bias the results because of confounding.

In conclusion, this study has found substantial incremental costs of recurrence in Medicare patients with stage IB to IIIA NSCLC, driven by an increased need for comprehensive health care services. New therapeutic options in the adjuvant setting such as ICIs and targeted therapies that decrease recurrence may reduce the subsequent economic burden of eNSCLC in this population.

## CRediT Authorship Contribution Statement

**Jay M. Lee:** Conceptualization, Formal Analysis, Investigation, Methodology, Supervision, Writing – reviewing & editing.

**Rongrong Wang:** Data Curation, Formal Analysis, Investigation, Methodology, Resources, Supervision, Validation, Writing – original draft, Writing – review & editing.

**Ann Johnson:** Conceptualization, Funding Acquisition, Investigation, Supervision, Visualization, Writing – review & editing.

**Sarika Ogale:** Formal Analysis, Investigation, Methodology, Resources, Supervision, Validation, Writing – original draft, Writing – review & editing.

**Matthew Kent:** Data Curation, Formal Analysis, Software, Validation, Visualization, Writing – review & editing.

**Janet S. Lee:** Conceptualization, Formal Analysis, Investigation, Methodology, Project Administration, Supervision, Writing – original draft, Writing – review and editing.
