# Supplementary figures and images for: Economic Burden of Recurrence Among Resected Medicare Patients With Early Stage NSCLC
Source: JTO Clin Res Rep. 2023 Feb 25;4(4):100487. doi: 10.1016/j.jtocrr.2023.100487 (PMC10050774; doi:10.1016/j.jtocrr.2023.100487)

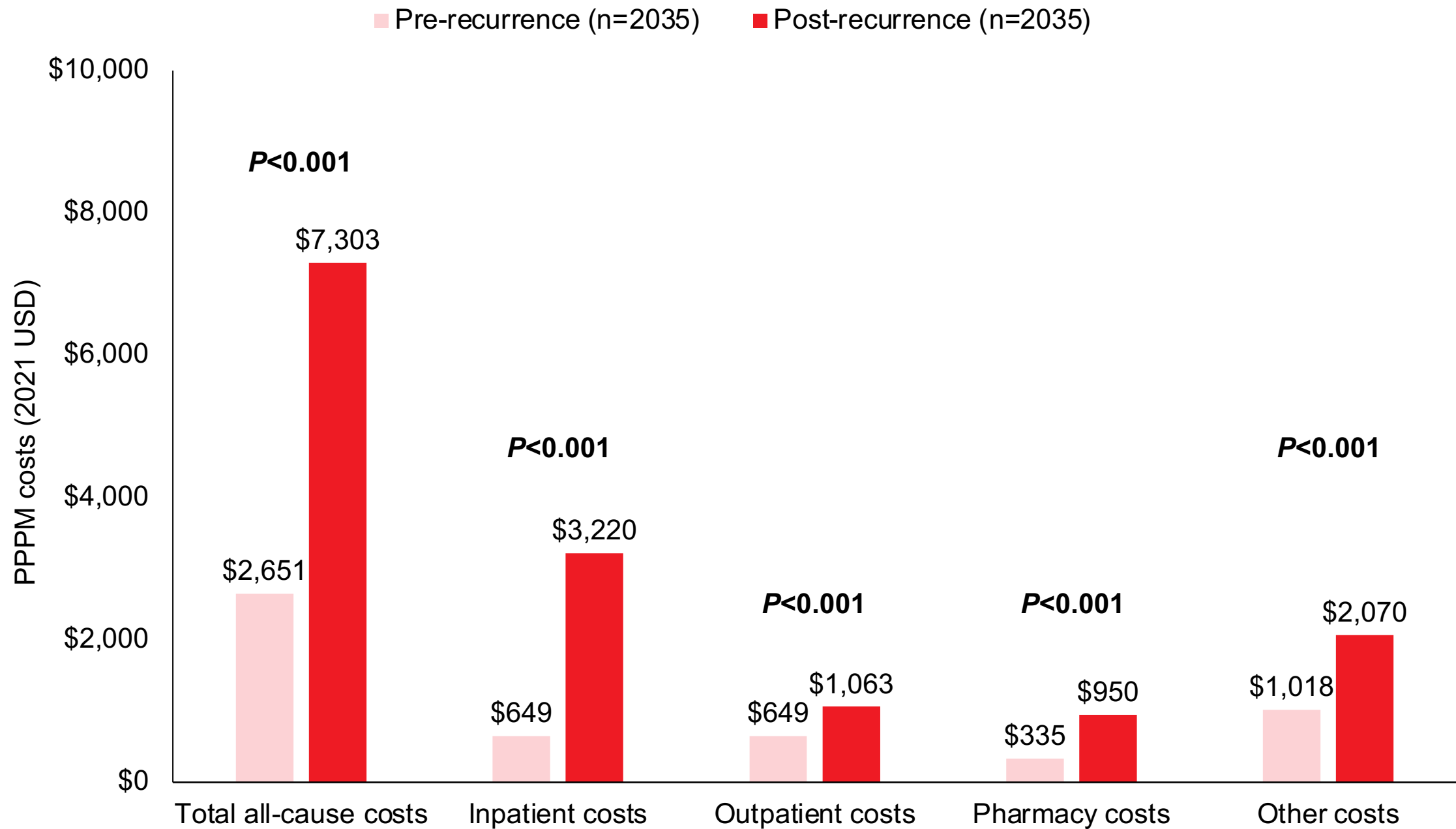

Supplement: Supplementary Data [file mmc1.pdf]
