# Supplementary material for: Economic Burden of Recurrence Among Resected Medicare Patients With Early Stage NSCLC
Source: JTO Clin Res Rep. 2023 Feb 25;4(4):100487. doi: 10.1016/j.jtocrr.2023.100487 (PMC10050774; doi:10.1016/j.jtocrr.2023.100487)
Supplement: Supplementary Material [file mmc2.docx]

Economic Burden of Recurrence Among Resected Medicare Patients With Early Stage Non-Small Cell Lung Cancer

Jay M. Lee, MD,^a^ Rongrong Wang,^b^ Ann Johnson,^b^ Sarika Ogale, PhD,^b^ Matthew Kent,^c^ Janet S. Lee, PharmD, PhD^b^

^a^David Geffen School of Medicine at UCLA, 10833 Le Conte Ave, Los Angeles, CA 90095; ^b^Genentech Inc, 1 DNA Way, South San Francisco, CA 94080; ^c^Genesis Research, 111 River St, Suite 1120, Hoboken, NJ 07030

Jay M. Lee, jaymoonlee@mednet.ucla.edu; Rongrong Wang, wang.rongrong@gene.com; Ann Johnson, johnson.ann@gene.com; Sarika Ogale, ogale.sarika@gene.com; Matthew Kent, kent.matthew@gene.com; Janet S. Lee, lee.janet@gene.com

Address for correspondence:

Jay M. Lee, MD

Surgical Director, Thoracic Oncology Program

Associate Professor of Surgery

David Geffen School of Medicine at UCLA

Los Angeles, CA 90095

Tel: 1-310-794-7333

E-mail: jaymoonlee@mednet.ucla.edu

**Supplemental Data 1.** Treatment definitions

| **Treatment type** | **Treatment** |
| --- | --- |
| Surgery | – |
| Extent of surgery | Lobectomy |
|  | Bilobectomy |
|  | Pneumonectomy |
|  | Segmentectomy |
|  | Wedge resection |
|  | Other |
| Surgery type | Video-assisted thoracic surgery |
|  | Robot-assisted thoracic surgery |
|  | Thoracotomy |
|  | Sternotomy |
| Radiation | – |
| Stereotactic body radiation therapy | – |
| Chemotherapy | Cisplatin, carboplatin, paclitaxel, albumin-bound paclitaxel (nab-paclitaxel, Abraxane), etoposide, docetaxel, gemcitabine, pemetrexed, vinorelbine |
| Immunotherapy | Durvalumab, pembrolizumab, atezolizumab, ipilimumab, nivolumab, avelumab |
| Targeted therapy | Bevacizumab, ramucirumab, erlotinib, afatinib, gefitinib, osimertinib, dacomitinib, necitumumab, crizotinib, ceritinib, alectinib, brigatinib, lorlatinib, entrectinib, dabrafenib, trametinib, selpercatinib, pralsetinib, capmatinib, larotrectinib, vemurafenib, cabozantinib, cetuximab |
| Ablation | – |

**Supplemental Data 2.** Incremental adaptation of recurrence rate definition

|  | **Definition 1** | **Definition 2** | **Definition 3** |
| --- | --- | --- | --- |
| **Criteria** | At least 1 inpatient claim or 2 outpatient claims with diagnosis codes for secondary malignant neoplasm (excluding lung) | - At least 1 inpatient claim or 2 outpatient claims with diagnosis codes for secondary malignant neoplasm (excluding lung) - **Surgery based on procedure codes** - **Non-surgical treatment (radiation, chemotherapy, immunotherapy, targeted therapy) based on procedure/drug codes** | - At least 1 inpatient claim or 2 outpatient claims with diagnosis codes for secondary malignant neoplasm (excluding lung) - Surgery based on procedure codes - Non-surgical treatment (radiation, chemotherapy, immunotherapy, targeted therapy) based on procedure/drug codes - **Diagnosis code for secondary malignant neoplasm of lung** - **Palliative care based on diagnosis codes** - **Biopsies based on procedure codes** |
| **5-Year recurrence rate, %** | 28 | 49 | 84 |
| **Recurrence events by stage, n (%)** | 1154 (25) | 2063 (45) | 3611 (79) |
| **IB** | 357 (18) | 700 (35) | 1493 (76) |
| **II** | 381 (25) | 712 (47) | 1203 (81) |
| **IIIA** | 416 (37) | 651 (58) | 915 (83) |

Hassett et al (2014)^13^ validation chart abstraction study of secondary malignant neoplasm and chemotherapy codes as indicators of recurrence after definitive local therapy for Stage I-III lung cancer using Cancer Care Outcomes Research and Surveillance Consortium (CanCORS)/Medicare and Health Maintenance Organization (HMO)/Cancer Research Network. To avoid chemotherapy given for adjuvant treatment, chemotherapy identified within 6 months of diagnosis was excluded.

Bold text indicates incremental changes to recurrence rate definitions.

**Supplemental Data 3.** HCRU in matched cohorts with Stage II-IIIA eNSCLC

| **HCRU, mean (SD), PPPM** | **Recurrence**  **(n=898)** | **No recurrence**  **(n=898)** | **Difference**  **(95% CI)** | ***P* value** |
| --- | --- | --- | --- | --- |
| Inpatient visit claims | 0.30 (0.47) | 0.09 (0.16) | 0.21  (0.18, 0.24) | <0.001 |
| Outpatient visit claims | 1.42 (1.56) | 0.59 (1.24) | 0.82  (0.69, 1.00) | <0.001 |
| Physician services claims | 4.70 (4.90) | 2.30 (3.30) | 2.50  (2.10, 2.90) | <0.001 |
| ED visit claims | 0.32 (0.45) | 0.10 (0.16) | 0.21  (0.18, 0.24) | <0.001 |

CI, confidence interval; ED, emergency department; eNSCLC, early non-small cell lung cancer; HCRU, health care resource use; PPPM, per patient per month; SD, standard deviation.

Recurrence based on Definition 2 in Supplemental Data 2.

**Supplemental Data 4.** Sensitivity analysis results: Pre- vs post-recurrence HCRU

| **HCRU, mean (SD), PPPM** | **Pre-recurrence**  **(n=2035)** | **Post-recurrence**  **(n=2035)** | **Difference**  **(95% CI)** | ***P* value** |
| --- | --- | --- | --- | --- |
| Inpatient visit claims | 0.35 (0.51) | 0.10 (0.09) | 0.26  (0.23, 0.28) | <0.001 |
| Outpatient visit claims | 1.78 (1.65) | 0.94 (0.87) | 0.85  (0.78, 0.91) | <0.001 |
| Physician services claims | 6.10 (5.10) | 3.00 (1.60) | 3.10  (2.90, 3.30) | <0.001 |
| ED visit claims | 0.31 (0.36) | 0.13 (0.13) | 0.18  (0.17, 0.20) | <0.001 |

CI, confidence interval; ED, emergency department; HCRU, health care resource use; SD, standard deviation.

Recurrence based on Definition 2 in Supplemental Data 2.

**Supplemental Data 5.** Sensitivity analysis results: Pre- vs post-recurrence all-cause healthcare costs


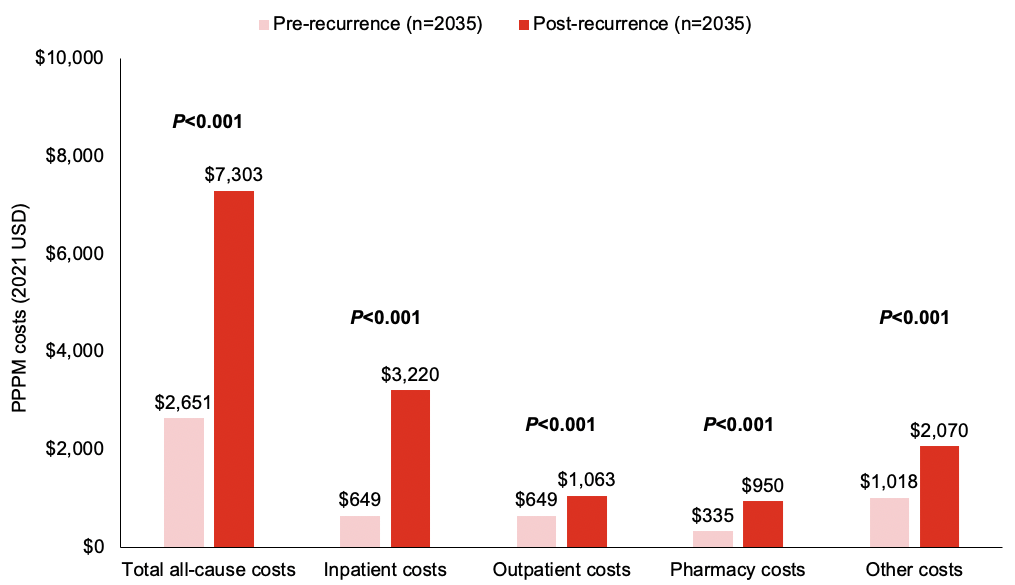


PPPM, per patient per month; USD, US dollars.

Recurrence based on Definition 2 in Supplemental Data 2.
